# Sustainable removal of malachite green from wastewater using mesoporous BaTiO₃: synthesis, characterization, and adsorption performance

**DOI:** 10.1186/s13065-025-01652-6

**Published:** 2025-11-06

**Authors:** Ahmed Barakat, Wael I. Mortada, A. M. Abdelghany, Magdi E Khalifa

**Affiliations:** 1 Chemistry Department, Faculty of Science, Mansoura University, Mansoura, 35516 Egypt; 2 Urology and Nephrology Center, Mansoura University, Mansoura, 35516 Egypt; 3 Spectroscopy Department, Physics Research Institute, National Research Centre, Giza, 12311 Egypt; 4 Basic and Applied Science Department, International Coastal Rd, Horus University, Kafr Saad, New Damietta, 34511 Egypt

**Keywords:** Barium titanate, Malachite green, Removal, Wastewater

## Abstract

In this study, mesoporous barium titanate (BaTiO₃) was synthesized using a single-step solid-state reaction by blending TiO₂ and BaCO₃ followed by thermal treatment. The produced BaTiO₃ served as an effective adsorbent for the uptake of malachite green (MG) from aqueous media. The synthesized BaTiO₃ was subjected to characterization using XRD, FTIR, SEM, TEM, EDX, and BET, demonstrating the formation of a crystalline, mesoporous perovskite structure with an average crystallite size of 68.2 nm and a surface area of 28.1 m²/g. Although its surface area is moderate, BaTiO₃ had a substantial maximum adsorption capacity of 495.15 mg/g, attributable to its oxygen vacancies, electrostatic attraction, and robust chemisorption mechanisms. The effect of various experimental factors, for example, pH, initial MG concentration, adsorbent dosage, contact time, temperature, and ionic strength, on MG removal using batch adsorption experiments was investigated. Optimum MG removal was achieved at pH of 5.0, adsorbent dose of 10.0 mg, initial solution volume of 10.0 mL, contact time of 330 min., and temperature of 50 °C. Kinetic studies were consistent with pseudo-second order model and the Langmuir model provided the best description of the adsorption isotherm. Thermodynamic variables such as (ΔG°, ΔH°, and ΔS°) were determined, and it was shown that the adsorption was feasible, spontaneous, and endothermic. Effective MG removal of over 98% was demonstrated in real sample applications comprising models of industrial wastewater and water from the Nile River. The study revealed that MG could be successfully extracted from wastewater samples using BaTiO_3_, which may be produced in a sustainable manner.

## Introduction

Synthetic dyes are environmentally harmful and non-biodegradable organic molecules, positioning them as significant industrial pollutants [1]. They are extensively utilized across multiple sectors such as textiles, paper, cosmetics, leather, printing, plastics, food, and medicines to add color to products. However, these dyes have adverse effects on the environment due to their toxicity to humans, as well as the ability to block photosynthetic phenomena in aquatic life by reducing light penetration [2, 3].

Malachite green (MG) is one type of organic cationic dye that dissolves in water and belongs to the triphenyl methane family. It is used in many applications in industry, including dyeing paper, leather, silk, and wool. Additionally, it is utilized as a disinfectant and fungicide in fish farms [4]. The liver, kidney, intestines, gonads, and pituitary glands are all severely harmed by MG dye [5, 6]. It is extremely harmful and highly cytotoxic to mammalian cells, which is why it is thought to be teratogenic, carcinogenic, mutagenic, toxic to the respiratory system, and impairs human fertility [2, 7]. MG is classified as a Class-II type toxin [8].

Physical, chemical, and advanced treatment techniques, like adsorption, membrane filtration, reverse osmosis, coagulation, flocculation, ion exchange, electrochemical technology, ozonation, chemical oxidation, and biological treatments, are among the common tools available to treat wastewater containing dyes [9–11]. Adsorption is the most attractive of such techniques due to its straightforward design, reusable adsorbents, simplicity of use, non-toxicity, affordability, and comparatively high efficiency [12].

The perovskite family can be assessed as adsorbent materials or catalysts for the removal of dyes [13]. Barium titanate (BaTiO₃) is a ferroelectric substance that is related to the ABO₃ perovskite structure family. The perovskite structure has two cations, Ba²⁺ and Ti⁴⁺, represented by the sites “A” and “B,” respectively, while the anion is oxygen (O), which forms bonds with both cations [14, 15]. Traditional methods for synthesizing BaTiO₃ include sol-gel, hydrothermal, and coprecipitation, which require the use of toxic solvents, a long time, or complex conditions. In this study, BaTiO₃ was prepared via the sol-gel approach, which is solvent-free, scalable, and energy-efficient [16].

BaTiO₃ has been considered a promising material for extensive applications, such as electronic device manufacturing, dynamic random access memory (DRAM) processing, high-frequency filters, and other integrated condenser structures [17, 18]. BaTiO₃ has a direct band gap of 3.20 eV and is utilized in transducers, thermistors, and electro-optical instruments [19]. The tetragonal perovskite structure of BaTiO₃ is characterized by ferroelectricity, low leakage current, and high dielectric permittivity at room temperature. These properties play a crucial role in adsorption and photocatalysis by influencing surface charge behavior, promoting electrostatic attraction between the adsorbent and pollutant molecules, and facilitating efficient charge separation during pollutant degradation [20].

Owing to its benefits, BaTiO₃ has gained significant interest for the efficient removal of hazardous organic pollutants from water and wastewater. Its environmental friendliness, low cost, and non-toxicity make it suitable for sustainable treatment applications. High structural stability, multiple crystal phases, and abundant oxygen vacancies enhance its adsorption capacity and photocatalytic activity by providing more active sites. In addition, its redox potentials and availability in a broad spectrum of sizes and morphologies further promote light-driven reactions, while the rapid migration of surface charge carriers minimizes recombination, thus increasing degradation efficiency [21].

There are different strategies to enhance the ability of BaTiO₃ to remove pollutants from wastewater. These involve modifying the size and morphology of the particles by changing synthesis temperatures and the reaction periods, loading with noble metal nanoparticles (NPs) such as Ag and Au to create plasmonic effects that enhance light absorption and charge separation, fabricating composites with other semiconductors (such as BaTiO₃/ZnO, BaTiO₃/TiO₂) or carbon-based materials (like BaTiO₃/graphene) to improve surface reactivity and stability, and doping with metals or non-metals to modulate electronic structure and increase active sites [22].

This study aims to explore mesoporous BaTiO₃, a perovskite-structured material, as an efficient adsorbent for the removal of MG dye from aqueous media. Unlike conventional adsorbents, BaTiO₃ offers high surface reactivity, environmental stability, and tunable morphology. Its unique properties, combined with high adsorption capacity and ease of preparation, make it a promising candidate for wastewater remediation. The work focuses on a simple, sustainable synthesis route and systematic evaluation of adsorption performance under various operational conditions.

## Experimental

### Chemicals

In this research, chemicals of analytical grade were utilized. Anatase titanium dioxide (TiO₂; purity of 99.8%) and barium carbonate (BaCO₃; purity of 99.9%) were supplied by Sigma-Aldrich (St. Louis, MO, USA). To prepare a stock solution of 1000 mg/L MG, 0.1 g of MG (Merck, Darmstadt, Germany) was dissolved in 100 mL of distilled water, and the required working solutions were obtained by subsequent dilution. Analytical grade NaOH (≥ 98%, El-Gomhouria, Egypt) and HCl (37%, analytical grade, El-Gomhouria, Egypt) were utilized to control the pH of solutions.

### Instrumentation

BaTiO₃ was characterized through a variety of instruments. XRD (X-ray diffraction) was analyzed by a benchtop powder X-ray diffractometer. FTIR (Fourier transform infrared) spectra were obtained with a Nicolet FTIR spectrophotometer. SEM (scanning electron microscopy) images were acquired on Jeol JSM-6510LV equipment fitted with EDX (energy dispersive X-ray), while TEM (transmission electron microscopy) images were acquired by a Jeol JEM-2100 instrument. The BET (Brunner-Emmett-Teller) surface area was examined by a Quantachrome NOVA Touch 4LX analyzer. The concentrations of MG were tested using the Unico Model 1200 spectrophotometer at 618 nm. The pH of solutions was determined using a pH meter (Adwa AD1030). A rotary orbital shaker (BIBBY Stuart Scientific-SO1) was used in batch experiments. A centrifuge (80 − 2 electric) is also used in experiments to accelerate phase separation.

### Methods

#### Synthesis of BaTiO₃ powder

**Fig. 1:**
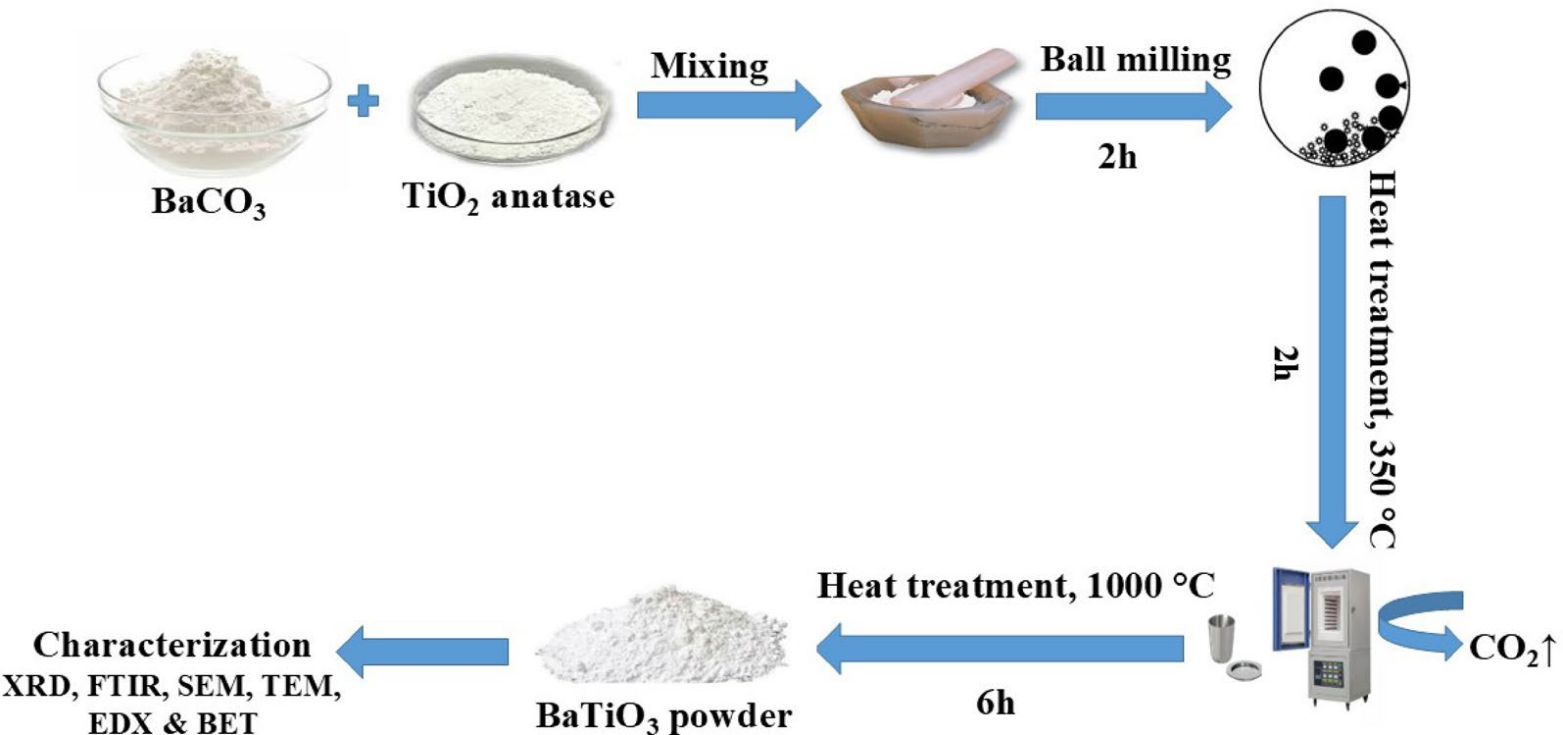
Schematic diagram of BaTiO₃ synthesis

BaTiO₃ was synthesized by a single-step solid-state reaction route [23]. Equimolar amounts of TiO₂ and BaCO₃ were blended in an agate mortar and subjected to ball milling for 2 h. The resulting mixture was transferred to a platinum crucible and underwent a thermal treatment schedule that involved 2 h at 350 °C to remove carbon dioxide from BaCO₃, followed by 6 h at 1000 °C. Figure 1 summarizes the steps for preparation of BaTiO₃.

#### pH_PZC_

pH_PZC_ is the pH at which the charge of the solid surface is neutral. To estimate pH_PZC_, individual flasks containing 50 mL of NaCl solution (0.1 mol/L), and the solutions were adapted to various pH (2–10) by NaOH (0.1 mol/L) and HCl (0.1 mol/L). Then, 20 mg of BaTiO₃ was added in each flask which were set in a shaker at 200 rpm. After 24 h, the BaTiO₃ was extracted from the solution, and the final pH of the solutions was determined. The change in pH (∆pH) was plotted versus the initial pH to estimate the pH_ZPC_. The pH_ZPC_ of BaTiO₃ is the point at which pH_initial_ = pH_final_.

#### Batch adsorption experiments

Batch adsorption tests were executed to assess the influences of experimental factors on MG removal using BaTiO₃. These factors include pH (4–10), adsorbent dose (5–40 mg), initial dye concentration (100–1000 mg/L), contact time (30–360 min), temperature (25–50 °C), and ionic strength (0.05–0.25 mol/L of NaCl). In a typical experiment, 10 mL of MG solution at the desired pH was mixed with a known quantity of BaTiO₃, and the solution was agitated by a shaker at 200 rpm at room temperature. After equilibrium time, the samples were centrifuged for 15 min. at 4000 rpm to separate BaTiO₃ from the solution, and then the dye concentration in the supernatant was estimated by spectrophotometer at 618 nm. The percentage of MG removal (%E) and the amount of MG adsorbed at equilibrium (q_e_) (mg/g) were determined by formulas (1) and (2), respectively:

1 $$\:\text{\%}\text{E}\:=\frac{\text{C}\text{0}-\text{C}\text{e}}{\text{C}\text{0}}\:\times\:100\:$$

2 $$\:\text{q}\text{e}\text{\:=\:}\frac{\left(\text{C}\text{0}-\text{C}\text{e}\right)\:\text{V}}{\text{m}}\:\:$$

Where C_0_ (mg/L) is the initial dye concentration, C_e_ (mg/L) is the equilibrium dye concentration, V (L) is the volume of solution, and m (g) is the mass of the adsorbent.

## Results and discussion

### Characterization of BaTiO₃

#### XRD

**Fig. 2:**
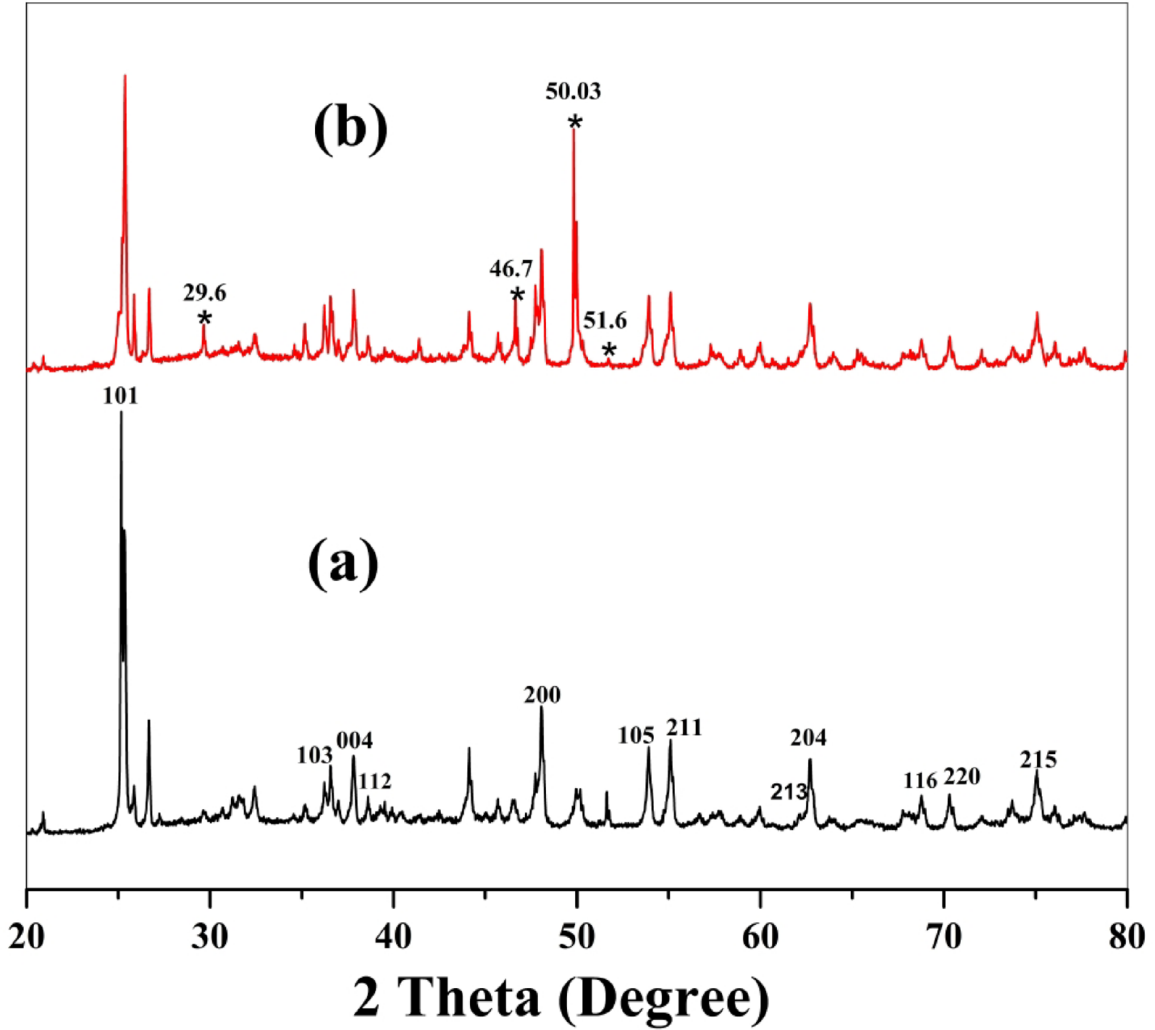
XRD pattern of BaTiO₃: **a** before adsorption of MG and **b** after adsorption of MG

Figure 2 indicates the XRD curves of BaTiO₃ before and after MG adsorption. According to Fig. 2a, there are diffraction peaks at 2θ = 25.3^◦^, 36.9^◦^, 37.8^◦^, 38.6^◦^, 48.1^◦^, 53.9^◦^, 55.1^◦^, 62.1^◦^, 62.7^◦^, 68.8^◦^, 70.3^◦^, and 75^◦^ with corresponding planes (101), (103), (004), (112), (200), (105), (211), (213), (204),(116), (220) and (215), respectively. Some of these peaks are consistent with the tetragonal BaTiO₃ phase (JCPDS No. 05-0626) [24]. The majority of peaks correspond to anatase TiO₂ (JCPDS No. 21-1272), which has lattice parameters a = b = 3.785 Å and c = 9.514 Å with the I41/amd space group [25]. This indicates the presence of unreacted precursor materials, which may result from an incomplete solid-state reaction during synthesis. Similar results have been documented in BaTiO₃ synthesized via solid-state methods, where residual secondary TiO₂ or Ba-rich compounds (such as BaCO₃ and Ba₂TiO₄) may occur if the calcination process is not sufficiently tuned [26, 27].

The average crystallite size of the particles can be determined using the Debye-Scherer formula (3).

3 $$\:\text{D}=\:\frac{\text{K}\:{\uplambda\:}}{{\upbeta\:}\text{cos}{\uptheta\:}}\:\:$$

Where D is the average crystallite size (nm), k is a constant (shape factor around 0.9), λ corresponds to the wavelength of the X-ray applied (0.15406 nm), β is the FWHM (full width at half maximum) of the diffraction lines, and θ is the Bragg diffraction angle. The average crystallite size of the particles was determined to be 68.22 nm.

In Fig. 2b, changes in some diffraction peaks were observed (additional peak at 2θ = 29.6°, peak disappeared at 2θ = 51.6°, and peaks intensity at 2θ = 46.7° and 50.03°). The peaks may be attributed to the interaction between MG dye and BaTiO₃ surface. The crystalline peaks of BaTiO₃ remain unchanged, indicating the integrity of BaTiO₃ and its potential for reuse in dye removal.

#### FTIR

**Fig. 3:**
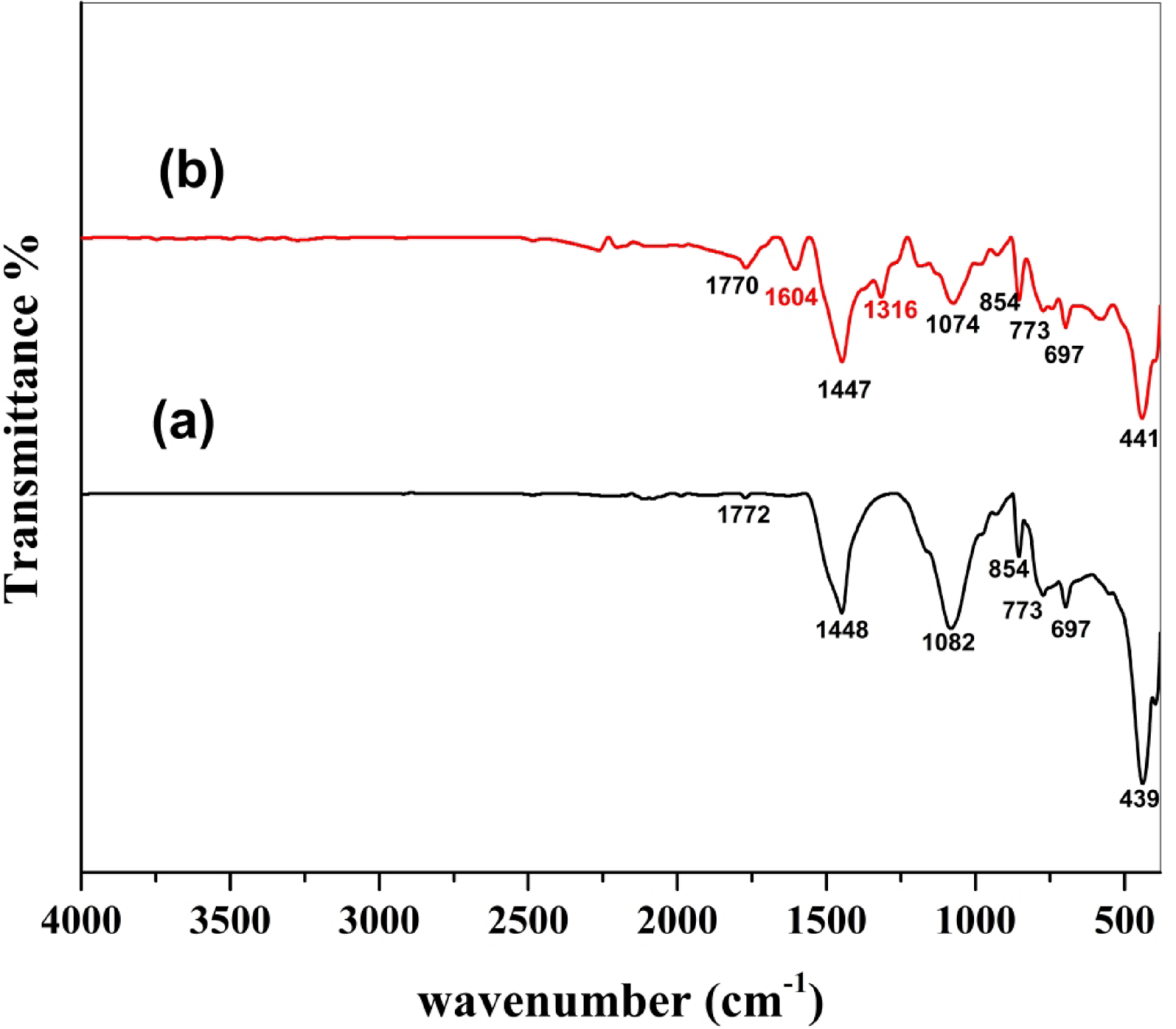
FTIR spectra of BaTiO₃: **a** before adsorption of MG and **b** after adsorption of MG

Infrared spectroscopy is a helpful method to recognize the functional groups that exist and decide more about the nature of possible interactions between BaTiO₃ and MG dye. Figure 3 presents FTIR spectra of BaTiO₃ before and after MG adsorption. The FTIR spectrum of BaTiO₃ powder displays peaks at 1772, 1448, 1082, 854, 773, 697, and 439 cm⁻¹. Whereas after adsorption of MG, the peaks were recorded at 1770, 1604, 1447, 1316, 1074, 854, 773, 697, and 441 cm⁻¹. The peak observed at 439 cm⁻¹ could be a feature of Ba-O stretching vibration [28]. The peaks at 697, 773, and 854 cm⁻¹ indicate the presence of Ti-O or Ti-O-Ti stretching vibrations [29]. In the FTIR spectra of BaTiO₃ after MG adsorption, new peaks appeared at 1316 and 1604 cm⁻¹ potentially ascribed to C-N stretching vibration and C = C stretching vibrations of aromatic rings, respectively, which are characteristic of the dye [30]. In addition, some peaks shifted slightly after adsorption (e.g., 439 → 441 cm⁻¹, 1082 → 1074 cm⁻¹, 1448 → 1447 cm⁻¹, and 1772 → 1770 cm⁻¹), and there was also a decrease in intensity for several peaks. These spectral changes, especially the appearance of new peaks and the shifts in peak positions, suggest interactions between BaTiO₃ and functional groups of MG dye and the successful attachment of MG molecules onto the BaTiO₃ surface. This supports that chemisorption was participating in the adsorption process.

#### SEM/EDX analysis

**Fig. 4:**
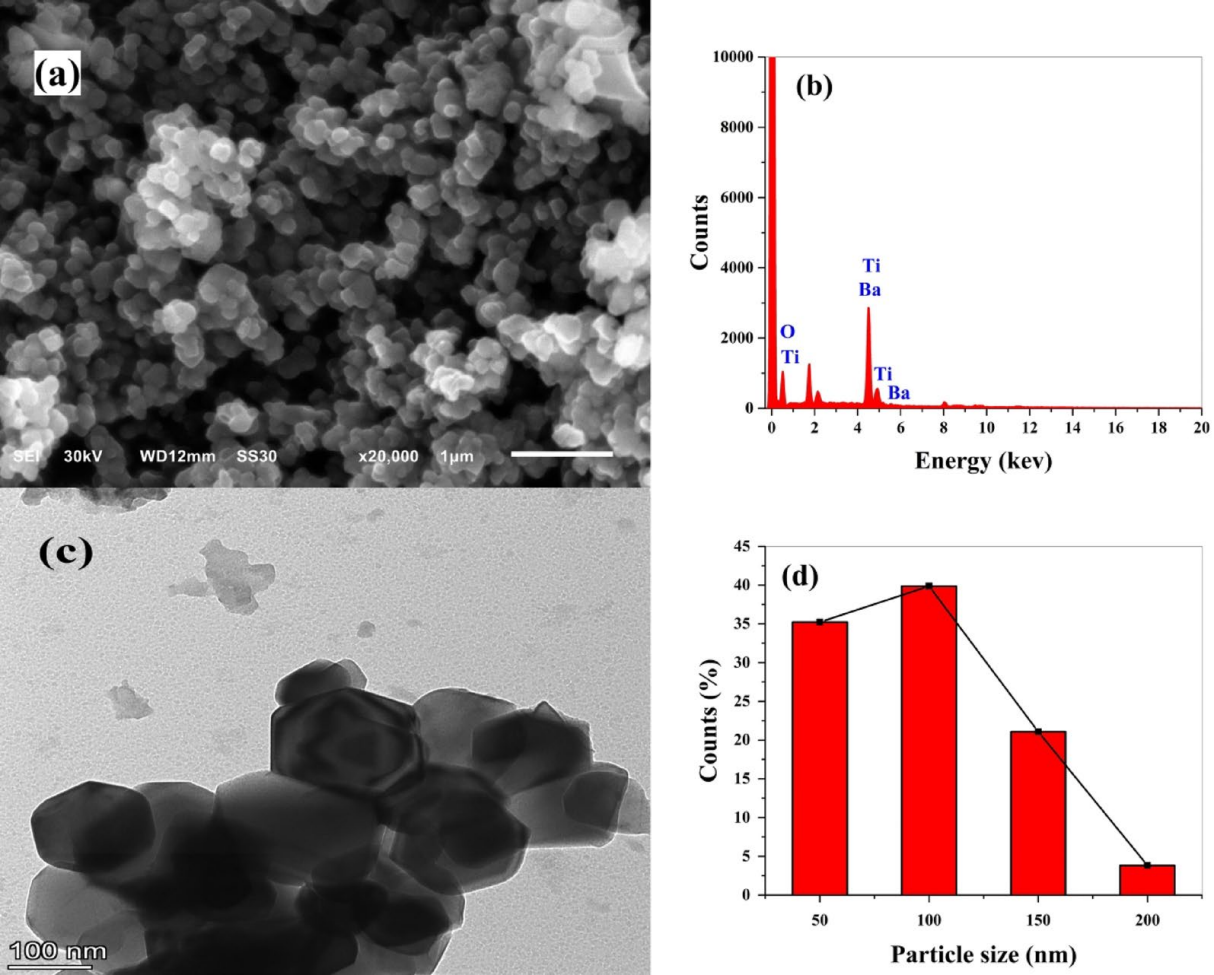
**a** SEM image; **b** EDX image; **c** TEM image; **d** Particle size distribution histogram; for BaTiO₃

SEM images are necessary to examine the surface morphology of the investigated sorbent. Figure 4a shows a SEM micrograph of the prepared BaTiO₃. The image illustrates that the particles are clusters of irregular spherical shape and uniformly distributed, which is a common feature in metal oxide nanomaterials. The surface morphology of BaTiO₃ microstructures looks to be dense and with clear grain boundaries. These grain boundaries and spaces between particles can serve as additional active sites for dye adsorption.

Energy dispersive X-ray is an analytical technique that determines the elemental components of a sample. The EDX spectrum confirmed the elemental composition of the BaTiO₃ sample, showing Ba, Ti, and O as the major constituents (Fig. 4b). The percentage mass of elements: barium (20.47%), titanium (31.83%), and oxygen (47.70%) are displayed in Table 1. This result confirms the successful synthesis of BaTiO₃ powder.

#### TEM

The TEM image (Fig. 4c) shows that BaTiO₃ is highly crystalline, and the particles are irregular spherical shapes with different particle sizes. The average particle size is 71.72 nm, as shown in the histogram of particle size distribution (Fig. 4d), which is consistent with the XRD calculations.

**Table 1:** EDX analysis of BaTiO₃

Element	Weight%	Atomic %
**O**	47.70	78.55
**Ti**	31.83	17.52
**Ba**	20.47	3.93
Total	100.0	100.0

#### BET studies

**Fig. 5:**
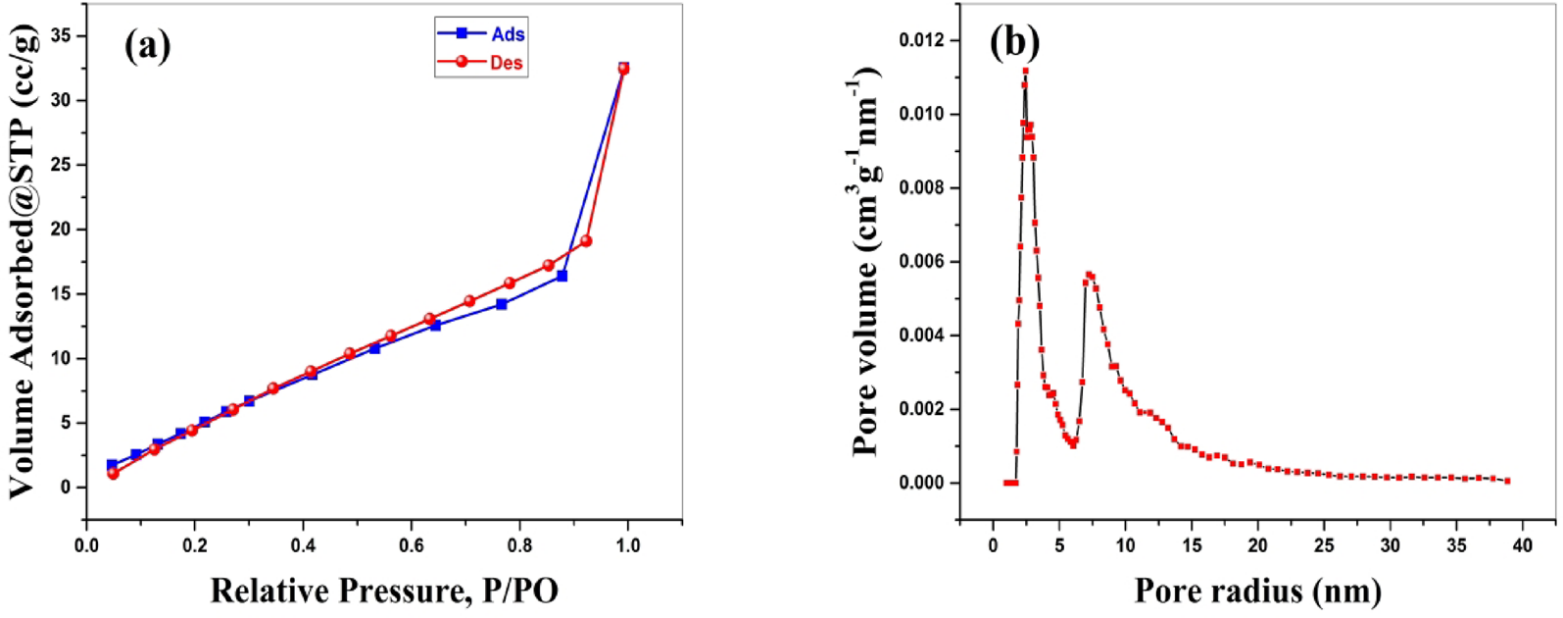
**a** N₂ adsorption-desorption isotherm; **b** BJH plot and pore size distribution

The surface area and pore characteristics for BaTiO₃ were established by utilizing gaseous N₂ adsorption-desorption isotherms at 77 K. Based on the IUPAC classification, the BaTiO₃ adsorption isotherm is of type IV. The existence of a hysteresis ring reveals that BaTiO₃ has a mesoporous structure, as depicted in Fig. 5a. The BET surface area of BaTiO₃ was detected to be 28.1 m²/g. Average pore radius and pore volume could be determined via the Barret-Joyner-Halenda (BJH) approach and observed to be 3.59 nm and 0.0437 cc/g, respectively. Figure 5b provides the corresponding pore size distribution curve. The mesoporous structure of BaTiO₃, characterized by pore sizes suitable for the accommodation of organic dye molecules, promotes efficient diffusion and interaction, which in turn improves the adsorption of MG dye.

#### Point of zero charge

**Fig. 6:**
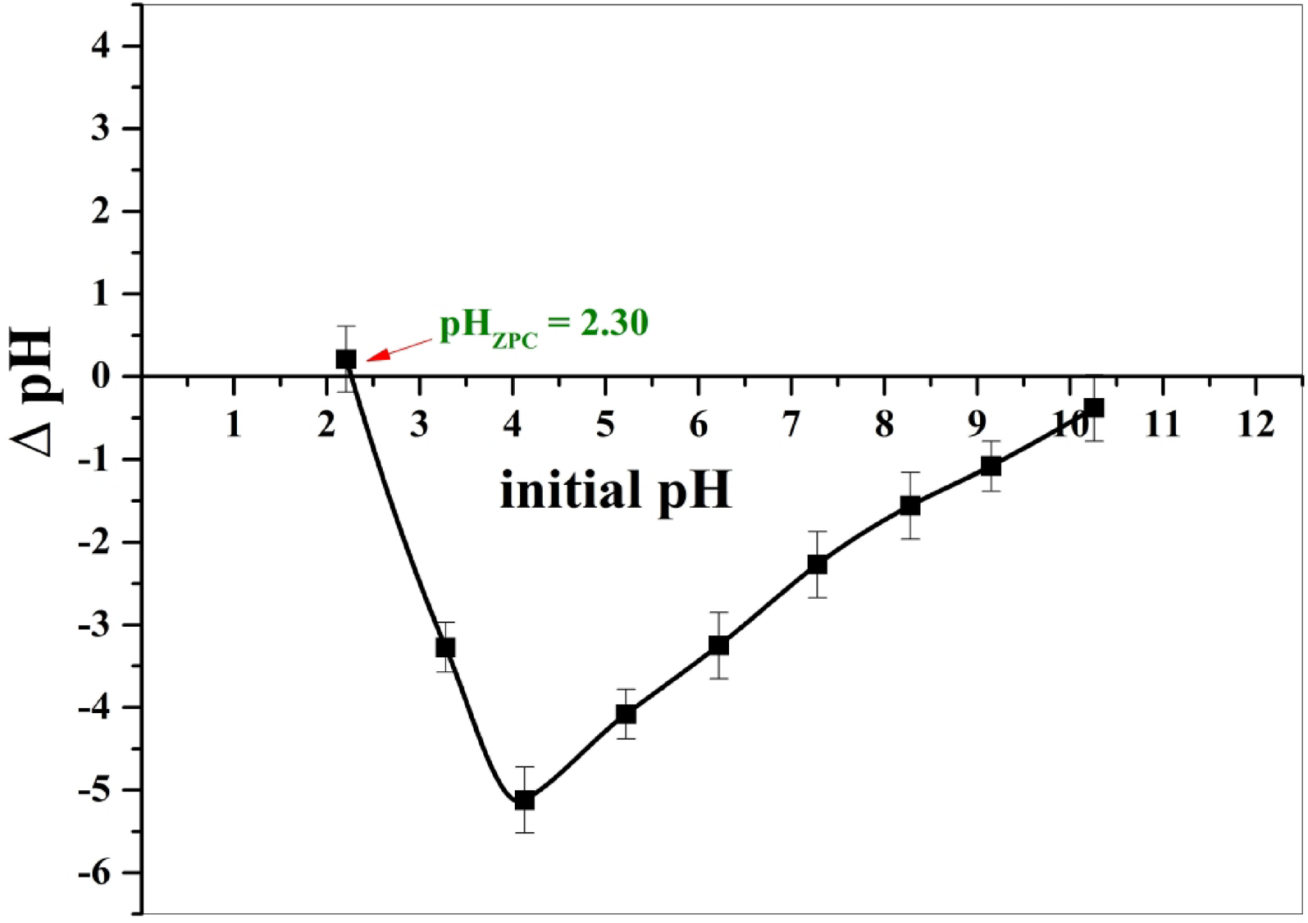
Point zero charge (PZC) of BaTiO₃

The pH_PZC_ was noticed to be achieved at pH 2.3 (Fig. 6). The pH_PZC_ of BaTiO₃ is lower than the values reported in the old literatures; this may be due to barium ions leached from BaTiO₃, which cause the particles of the surface to be enriched in TiO₂ [31, 32].

### Batch adsorption process

#### Influence of pH

**Fig. 7:**
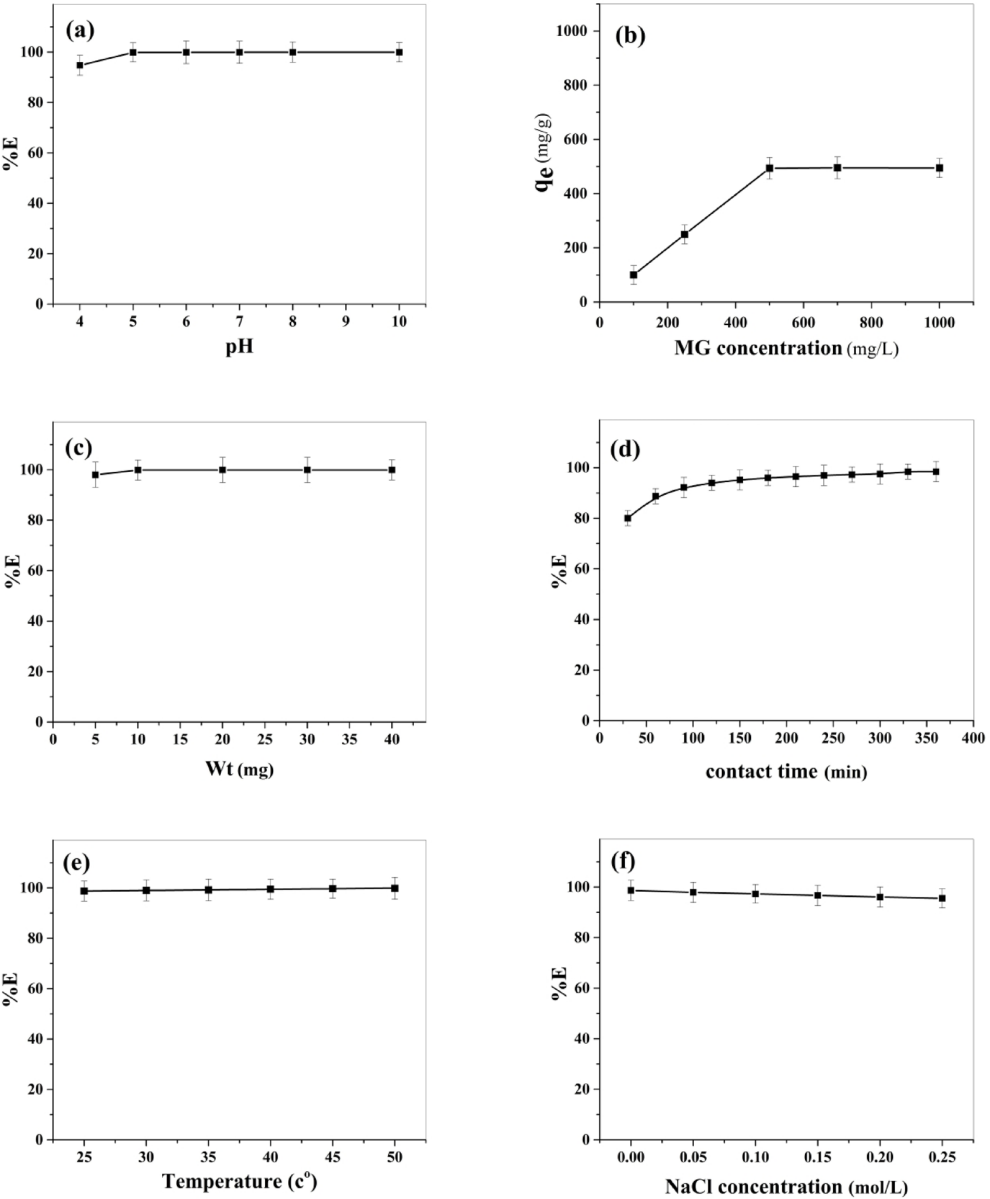
Influence of experimental parameters on MG removal by BaTiO₃: **a** pH (*Conditions*: initial dye concentration = 250 mg/L, adsorbent dose = 10 mg, volume = 10 mL, temperature = 25 °C, equilibrium time = 330 min); **b** initial dye concentration (*Conditions*: adsorbent dose = 10 mg, volume = 10 mL, temperature = 25 °C, equilibrium time = 330 min, pH = 5); **c** adsorbent dose (*Conditions*: initial dye concentration = 250 mg/L, volume = 10 mL, temperature = 25 °C, equilibrium time = 330 min, pH = 5); **d** contact time (*Conditions*: initial dye concentration = 500 mg/L, volume = 10 mL, temperature = 25 °C, equilibrium time = 330 min, pH = 5); **e** temperature (*Conditions*: initial dye concentration = 500 mg/L, adsorbent dose = 10 mg, volume = 10 mL, equilibrium time = 330 min, pH = 5); **f** ionic strength (*Conditions*: initial dye concentration = 500 mg/L, adsorbent dose = 10 mg, volume = 10 mL, pH = 5, temperature = 25 °C, equilibrium time = 330 min)

Generally, in adsorption experiments, the initial pH of a solution is one of the most crucial parameters affecting the adsorption efficiency, since it has an influence on the solubility, surface charge, adsorbate speciation, and ionization degree of adsorbate [33]. Therefore, the influence of pH on MG adsorption by BaTiO₃ over a pH range (4–10) was studied. The pH was adjusted by 0.1 mol/L NaOH or 0.1 mol/L HCl, and the other experimental factors such as initial dye concentration, adsorbent dose, contact time, and temperature were kept constant at 250 mg/L, 10 mg, 330 min, and 25 °C, respectively. Figure 7a represents the initial pH influence on the MG removal (%E). The figure illustrates that the removal of MG rises rapidly from 94.7% to 99.9% with a rise in pH from 4 to 5. At pH ranges from 5 to 10, the dye removal was almost fixed (99.9%). These findings can be interpreted regarding pH_ZPC_. At pH < pH_PZC_, the BaTiO₃ surface gains a positive charge, and the H⁺ concentration is higher, and thus competition with MG cations occurs for empty adsorption sites, resulting in a decline of dye uptake. At pH > pH_PZC_, the adsorbent surface becomes negatively charged and enhances cationic dye uptake because of electrostatic attraction force. Therefore, adsorption experiments were conducted at pH 5. At this pH, MG dye remains stable in its cationic form (MG⁺), exhibiting its characteristic green color [34].

#### Influence of initial concentration of MG

The influence of MG concentration on the efficiency of adsorption was investigated in the initial concentration range (100–1000) mg/L, keeping other conditions constant (adsorbent dose: 10 mg, volume: 10 mL, pH: 5, contact time: 330 min, and temperature: 25 °C). According to Fig. 7b, as the initial MG concentration rises from 100 to 500 mg/L, the adsorption capacity rises from 99.9 to 493.5 mg/g. This trend could be demonstrated through powerful mass transfer driving forces observed at high concentrations. Additionally, more dye molecules surround the adsorbent’s active sites when the dye concentration in the solution is high, which promotes more effective adsorption [35]. Once the dye concentration reaches 500 mg/L, no additional dye molecule uptake occurs. This could be interpreted as saturation of all available active sites of the adsorbent and the presence of electrostatic repulsion force between the dye on the surface of the adsorbent and the dye molecules in solution [36].

#### Influence of adsorbent dosage

To study the impact of adsorbent amount, the adsorption experiment was executed by changing adsorbent amount from 5 to 40 mg at constant initial dye concentration (250 mg/L), pH (5), contact time (330 min), and temperature (25 °C). As presented in Fig. 7c, when adsorbent dosage increases, the removal of MG percent also increases until it reaches a constant limit of 10 mg. Additionally, the figure illustrated that the dye uptake increased slightly from 98.0 to 99.9% when the dose increased from 5 to 10 mg. This is ascribed to a more extensive surface area and a greater number of adsorption sites [37]. Further addition of BaTiO₃ did not yield a significant increase in dye removal efficiency, as the percentage remained almost unchanged. This is due to saturation of the surface of BaTiO₃. So, for this reason, 10 mg of BaTiO₃ powder was chosen for further experiments.

#### Influence of contact time

The influence of contact time on dye adsorption was carried out with different intervals of time (30–360 min) at constant initial dye concentration 500 mg/L, adsorbent amount 10 mg, volume 10 mL, and pH 5 at temperature 25 °C, and the findings are displayed in Fig. 7d. As shown, the rate of adsorption is quite fast at first, and 80% of adsorption is accomplished during the first 30 min. A further increase in contact time (30–330 min) shows a slight increase in adsorption (80–98.5% dye removal). After 330 min, no change in the removal percentage was observed. Thus, the ideal contact time was regarded as 330 min. The rapid equilibrium is owing to the available number of active sites at the BaTiO₃ surface in the beginning stages of adsorption. Over time, the number of available active sites decreased continually until saturation was reached.

#### Influence of temperature

Temperature was another essential factor that possessed a direct effect on dye adsorption. It is vital to measure parameters of thermodynamics such as standard enthalpy change (∆H^o^), standard Gibbs free energy change (∆G^o^), and standard entropy change (∆S^o^). The influence of temperature on adsorption was investigated at 25, 30, 35, 40, 45, and 50 °C under ideal circumstances (10 mg of adsorbent to 10 mL of dye solution at pH 5, initial concentration = 500 mg/L, and contact time = 330 min). Figure 7e demonstrates that as the temperature increases, the removal of MG enhances. As the temperature rises from 25 to 50 °C, the removal increases from 98.7 to 99.8%, illustrating that the process of adsorption favors high temperatures and is endothermic in nature. By increasing the temperature, there is an increase in dye mobility [38].

#### Influence of ionic strength

Because wastewater typically contains larger concentrations of salt, it is important to investigate how ionic strength affects dye adsorption. The influence of ionic strength on MG removal by BaTiO₃ was examined under various NaCl concentrations (0.05–0.25 mol/L) at constant experimental conditions (initial concentration of dye: 500 mg/L, adsorbent dosage: 10 mg, volume: 10 mL, contact time: 330 min, temperature: 25 °C, and pH: 5). The findings in Fig. 7f show that the dye removal decreases slightly from 97.9 to 95.6% with rising concentrations of NaCl solution from 0.05 to 0.25 mol/L, respectively. This might be related to the competition impact between Na⁺ and cationic MG for the available active sites of the BaTiO₃ surface, leading to electrostatic repulsion, causing the adsorption process’s efficiency to decrease [39].

#### Adsorption kinetics

To expect the rate of adsorption and recognize the process’s controlling mechanism, adsorption kinetics is used in adsorption research. The adsorption process may be explained in four phases. They are: (i) Diffusion of MG dye from the bulk of the solution to the liquid film around BaTiO₃ particles; (ii) Diffusion of MG dye throughout the surface liquid film around BaTiO₃ particles; (iii) Intraparticle diffusion of MG dye involving pore diffusion and surface diffusion; and (iv) Interaction with active sites of the surface via physisorption or chemisorption. The slowest of the four phases above controls the overall rate of adsorption [40]. The behavior of MG adsorption onto the BaTiO₃ powder was investigated in this work using pseudo-first-order, pseudo-second order, and intra-particle diffusion models.

##### Pseudo-first-order kinetics

The rate of change of solute uptake over time is directly proportional to the variation in saturation concentration and the amount of solid uptake over time, according to the Lagergren pseudo-first-order model. This model is stated using formula (4).

$$\:\text{log\:}\left(\text{q}\text{e}-\text{q}\text{t}\right)=\text{log}\text{q}\text{e}-\left(\frac{\text{k}\text{1}}{2.303}\right)\text{t}\:\:\:\:\:\:\:\:\:\:\:\:\:\:\left(4\right)$$

**Fig. 8:**
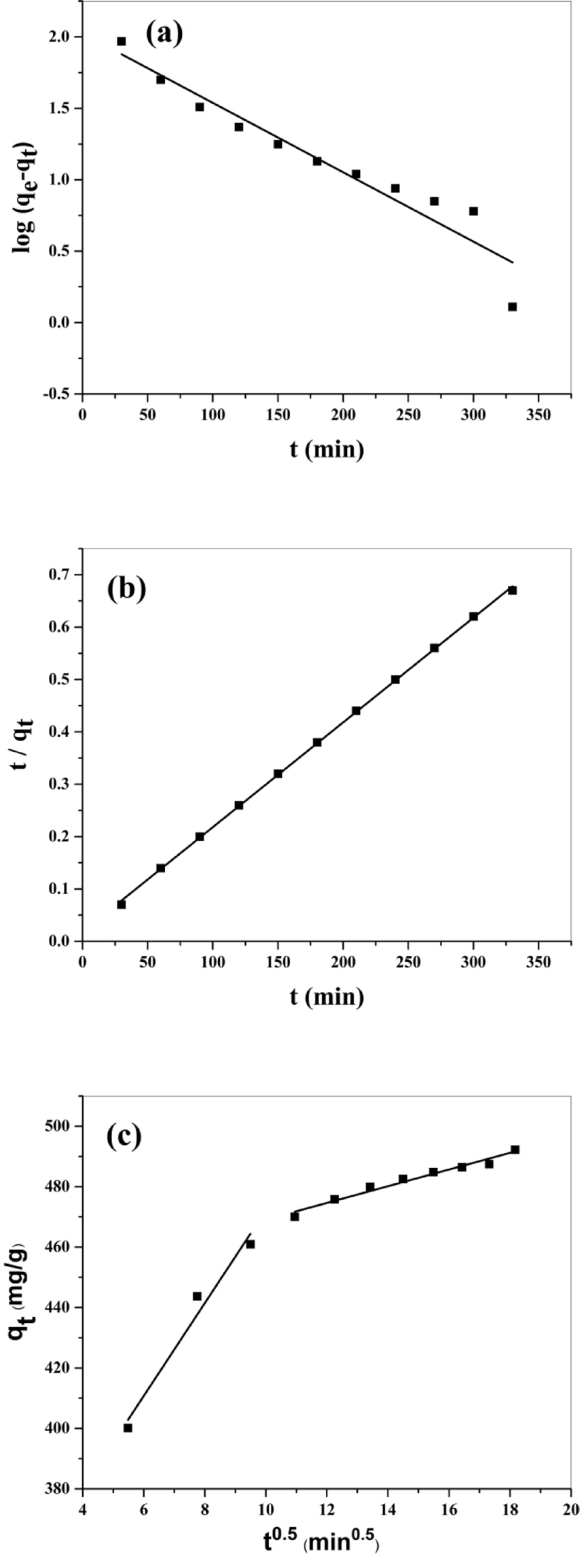
**a** Pseudo-first-order model; **b** Pseudo-second-order model; ( Intra-particle diffusion model for the adsorption of MG by BaTiO₃

Where q_e_ and q_t_ (both in mg/g) are, respectively, the adsorbed amounts at equilibrium and at time t, and k_1_ (min⁻¹) is the equilibrium rate constant for the pseudo-first-order adsorption. The magnitudes of k_1_ and q_e_ were measured from the slope and intercept of the linear plot of log (q_e_-q_t_) against t (Fig. 8a).

##### Pseudo-second-order kinetics

The pseudo-second-order model demonstrates chemisorption as well as cation exchange reactions. This model is defined using formula (5).

$$\:\frac{\text{t}}{\text{q}\text{t}}\:=\:\frac{1}{\text{k}\text{2}\text{q}\text{e}\text{2}}+\:\frac{\text{t}}{\text{q}\text{e}}\:\:\:\:\:\:\:\:\:\:\:\:\:\:\:\:\left(5\right)$$

Where k_2_ (g/mg.min) is the equilibrium rate constant of the pseudo-second-order adsorption and q_e_ was calculated from the plot of$\:\:\:\frac{\text{t}}{\text{q}\text{t}}$ versus t (Fig. 8b).

##### Intra-particle diffusion model

To estimate the rate-determining step, the intraparticle diffusion model was set to the study data. The formula can be written as

$$\:\text{q}\text{t}\:=\text{k}\text{id}{\text{t}}^{\raisebox{1ex}{$1$}\!\left/\:\!\raisebox{-1ex}{$2$}\right.}+\:\text{C}\text{i}\:\:\:\:\:\:\:\:\:\:\:\:\:\:\left(6\right)\:$$.

Where q_t_ (mg/g) is the adsorption capacity at time t (min), k_id_ (mg/g. min^0.5^) is the intra-particle diffusion constant, and C_i_ is the intercept at stage i and represents boundary layer thickness. k_id_ and C_i_ were computed from the (q_t_ against t^1/2^) plot. As noticed in Fig. 8c, there are two steps included in the adsorption of MG using BaTiO₃ powder. The first step is the external mass transfer (boundary layer diffusion); in this stage, the removal rate of MG is greater because of the instantly available large surface area and large active adsorption sites. The second step corresponded to the intraparticle diffusion effect. The R^2^ magnitudes are located in the range 0.94–0.96 of the linear plots, suggesting its usefulness to describe the adsorption process. The magnitude of K_1d_ (15.35 mg/g min^0.5^) is greater than K_2d_ (2.76 mg/g min^0.5^), indicating that the external diffusion principally influences the adsorption kinetics. In addition, the plot did not traverse the origin (C_i_ did not equal zero), proving that the intraparticle diffusion adsorption was not the rate-determining step.

Table 2 shows a comparison of the results obtained from correlation coefficients (R^2^) and calculated (q_e_) for pseudo-first-order kinetic and pseudo-second-order kinetic models. Clearly, the coefficient resulting from the pseudo-second-order model is greater than that of the pseudo-first-order model. Furthermore, the experimental adsorption capacity (493.5 mg/g) is matched with the calculated adsorption capacity (500 mg/g) from the pseudo-second-order model. This suggests the pseudo-second-order model could be applied to explain MG adsorption onto BaTiO₃ powder, and the adsorption process is chemisorption. Chemical reaction may occur by exchanging or sharing electrons between MG and BaTiO₃.

**Table 2:** Kinetic adsorption parameters for adsorption of MG onto BaTiO₃ adsorbent

Kinetic model	Parameters	Values
	q_e_ exp (mg/g)	493.5
Pseudo first order	R^2^	0.9156
	q_e_ calc (mg/g)	105.8
	k_1_ (min^− 1^)	1.11 × 10^− 2^
Pseudo second order	R^2^	0.9995
	q_e_ calc (mg/g)	500.0
	k_2_ (g/mg min)	2.2 × 10^− 4^
Intra particle diffusion	R^2^	0.9432
	K_1D_ (mg/g min^0.5^)	15.35
	C_1_ (mg/g)	318.7
	R^2^	0.9647
	K_2D_ (mg/g min^0.5^)	2.76
	C_2_ (mg/g)	441.5

#### Adsorption isotherms

Adsorptions Isotherms are essential for identifying the manner of interaction of adsorbate molecules with the surface of the adsorbent and to recognize the nature of the interaction. The most widely used isotherms are Langmuir, Freundlich, and Temkin models.

##### Langmuir isotherm

The Langmuir isotherms depend on the idea of monolayer adsorption onto an adsorbent surface consisting of a limited number of adsorption sites of consistent adsorption energies. It can be stated by a formula:

$$\:\frac{\text{C}\text{e}}{\text{q}\text{e}}=\:\frac{1}{\text{b}\text{q}\text{m}}+\:\frac{1}{\text{q}\text{m}}\:\text{C}\text{e}\text{\:\:\:\:\:\:\:\:\:\:\:\:\:\:\:\:(7)}$$

**Fig. 9:**
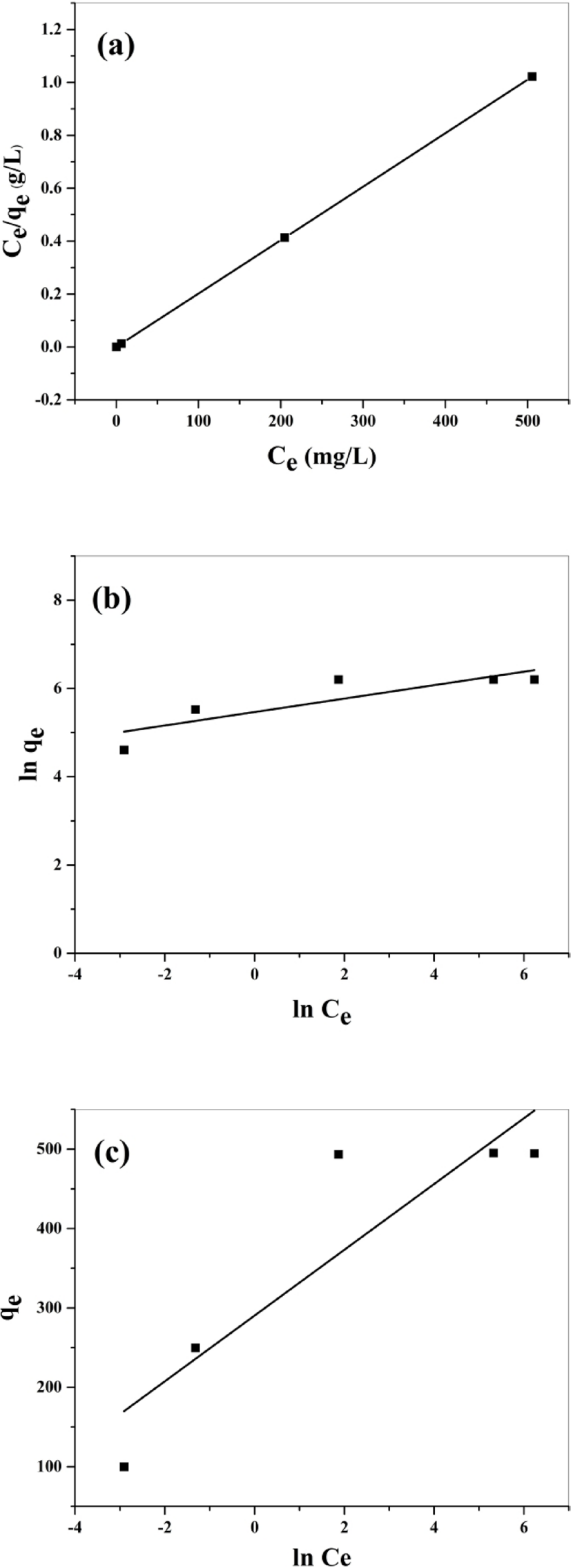
**a** Langmuir plot of MG adsorption onto BaTiO₃; **b** Freundlich plot of MG adsorption onto BaTiO₃; **c** Temkin plot of MG adsorption onto BaTiO₃

Where C_e_ (mg/L) is the dye concentration at equilibrium, q_e_ (mg/g) is the quantity of adsorbed dye at equilibrium, b (L/mg) denotes the Langmuir isotherm constant concerning the free energy of adsorption, and q_m_ (mg/g) is monolayer adsorption capacity. The magnitudes of q_m_ and b can be estimated from the slope and intercept of the linear plot of C_e_/q_e_ against C_e_ (Fig. 9a), respectively, and are displayed in Table 3; the correlation coefficient (R^2^) magnitude is also depicted. The Langmuir isotherm is categorized by a dimensionless constant separation factor (R_L_) to estimate if the process of adsorption is favorable or unfavorable for the Langmuir model. It can be written as:

$$\:\text{R}\text{L\:=\:}\frac{1}{1+\text{b}\text{C}\text{0}}\:\:\:\:\:\:\:\:\:\:\:\:\:\:\:\:\:\:\:\:\left(8\right)$$

Where b (L/mg) is the Langmuir isotherm constant and C_0_ (mg/L) is the highest initial concentration of dye. When R_L_*>*1, the adsorption is defined as unfavorable. When R_L_ = 1, the adsorption is linear, and when 0 *<* R_L_*<*1 implies that it is favorable.

##### Freundlich isotherm

The Freundlich isotherm reflects a multilayer adsorption capacity and predicts that the adsorption process happens on heterogeneous surfaces characterized by irregular heat distribution over the surface of the adsorbent. The Freundlich model can be presented by the linearized formula as:

$$\:\text{ln}\text{q}\text{e}\text{\:=}\text{ln}\text{K}\text{f}\text{+\:}\frac{1}{\text{n}}\text{ln}\text{C}\text{e}\text{\:\:\:\:\:\:\:\:\:\:\:\:\:\:(9)}$$

Where K_f_ and n are Freundlich constants, respectively concerned with adsorption capacity and adsorption intensity. The magnitudes of the K_f_ and n were estimated from the interception and slope of the (ln q_e_ against ln C_e_) linear plot (Fig. 9b), respectively, and are displayed in Table 3; the correlation coefficient (R^2^) is also given. The magnitude of 1/n reflects the nonlinear relationship between adsorption and solution concentration and should be less than one for the adsorption to be favorable.

##### Temkin isotherm

The Temkin isotherm takes into consideration the impact of indirect interaction between adsorbent and adsorbate molecules. Temkin observed that, as the coverage on the surface rises, the heat of adsorption for all the surface molecules reduces linearly. The linearized formula of the Temkin model is defined as:

$$\:\text{q}\text{e}\text{\:=\:}\frac{\text{R}\:\text{T}}{\text{b}\text{T}}\text{ln}\text{K}\text{T}+\frac{\text{R}\:\text{T}}{\text{b}\text{T}}\text{ln}\text{C}\text{e}\:\:\:\:\:\:\:\:\:\:\:\:\:\:\:\left(10\right)$$

Where R is the universal gas constant, T is the temperature, b_T_ is the Temkin constant concerning heat of adsorption, and K_T_ is the Temkin parameter concerning the equilibrium binding energy. From the plot of q_e_ against ln C_e_ (Fig. 9c), b_T_ and K_T_ can be estimated, respectively, from the slope and intercept of the plot and are displayed in Table 3; also, the correlation coefficient (R^2^) is determined.

As shown in Table 3, the experimental data confirmed that the magnitudes of the correlation coefficient were better for the Langmuir model (R^2^ = 0.9980) in contrast to the Freundlich model (R^2^ = 0.6654) and Temkin model (R^2^ = 0.7605). Consequently, the Langmuir model is the best to identify the adsorption process. The R_L_ value (8.431 × 10^− 5^) was located between 0 and 1, which suggests that MG dye adsorption using BaTiO₃ powder is favorable. The maximum monolayer adsorption capacity for the Langmuir model (q_max_ = 495.04 mg/g) appeared to be very near the experimental q_max_ (495.15 mg/g). Thus, a well-fit Langmuir isotherm reveals MG adsorbed as a monolayer on the BaTiO₃ surface. The adsorption data according to the Langmuir model suggest monolayer distribution of dye molecules on a uniform surface, aligning with the morphological and structural findings derived from characterisation experiments. SEM and TEM micrographs demonstrated a relatively uniform surface texture with evenly dispersed active sites, corroborating the assumption of homogeneity characteristic of the Langmuir model. Additionally, XRD patterns validated the crystalline structure and stability of the adsorbent, confirming the presence of ordered sites for dye adsorption. Collectively, these results indicate that the structural integrity and surface uniformity shown by SEM, TEM, and XRD investigations align well with the Langmuir adsorption behavior, thereby corroborating the model’s prediction of monolayer adsorption on a homogeneous surface.

**Table 3:** Isotherm parameters obtained for different models

Isotherm	Parameters	Values
Langmuir	R^2^	0.9980
	q_max_ (mg/g)	495.04
	B (L/m*g*)	11.859
	R_L_	8.431 × 10^− 5^
Freundlich	R^2^	0.6654
	K_f_	236.006
	*n*	6.56
Temkin	R^2^	0.7605
	K_T_ (L/g)	1107.72
	b_T_ (J/mol)	59.83

#### Thermodynamic studies

The thermodynamic variables, including Gibbs free energy change (ΔG°), change in enthalpy (ΔH°), and change in entropy (ΔS°), were determined for more recognition of how temperature affects the process of adsorption. The Gibbs free energy of adsorption ΔG^o^ can be determined from the next formula:

11 $$\:\varDelta\:\text{G}^\circ\:=\:-\text{R}\text{T}\:\text{ln}\text{K}\text{C}$$

Where R is the gas constant (8.314 J/mol K), T (K) is temperature, and K_C_ is the adsorption equilibrium constant. The magnitude of K_C_ is calculated from the next formula:

12 $$\:\text{K}\text{C}=\:\raisebox{1ex}{$\text{q}\text{e}$}\!\left/\:\!\raisebox{-1ex}{$\text{C}\text{e}$}\right.\:$$

Where q_e_ (mg/g) is the quantity of MG uptake at equilibrium, whereas C_e_ (mg/L) is the concentration of MG remaining in solution at equilibrium. Standard enthalpy and standard entropy of adsorption are easily determined by the Van’t Hoff formula:

13 $$\:\text{ln}\text{K}\text{C}=\:-\:\frac{\varDelta\:\text{H}^\circ\:}{\text{R}\text{T}}+\:\frac{\varDelta\:\text{S}^\circ\:}{\text{R}}$$

**Fig. 10:**
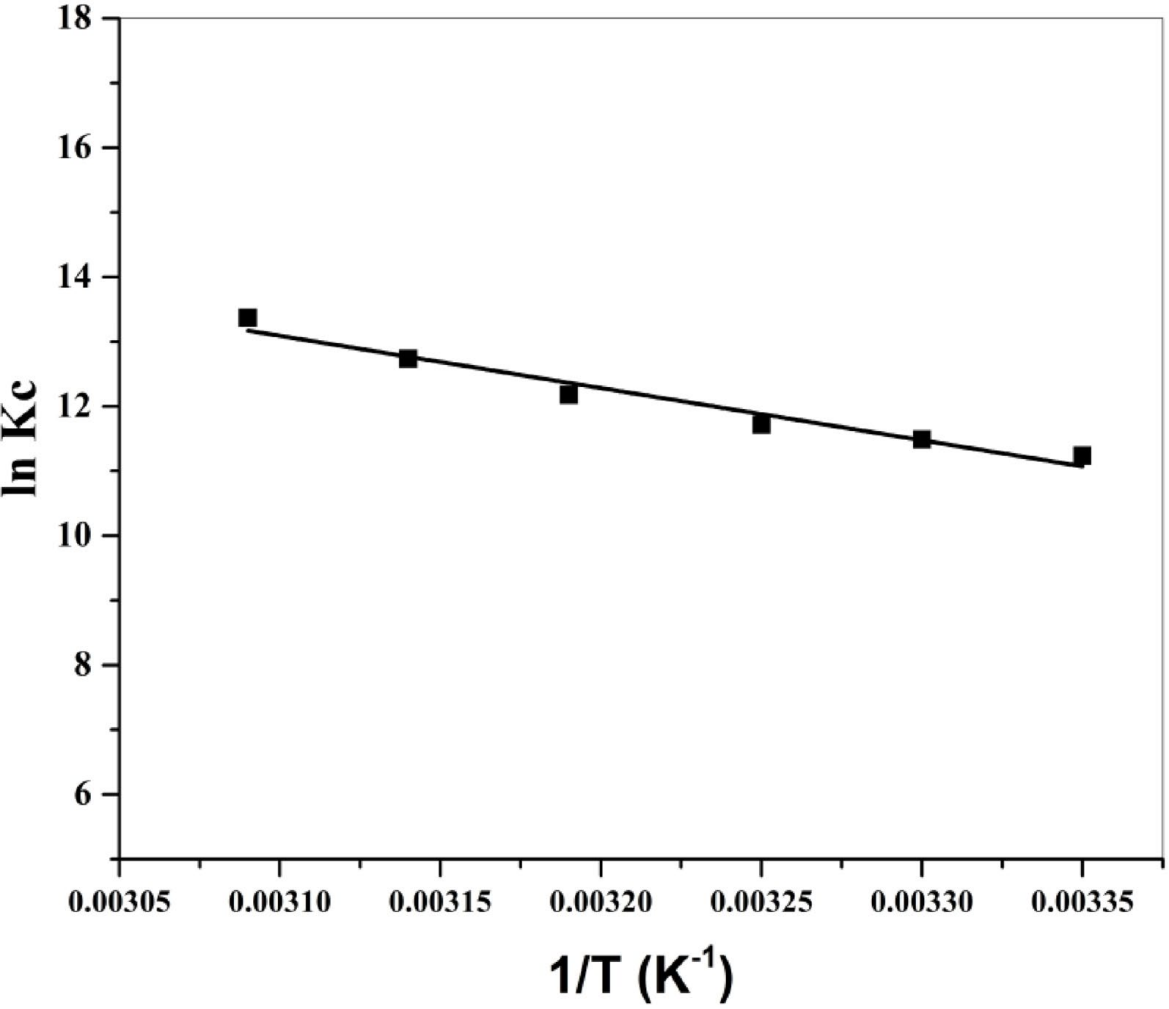
Van’t Hoff plot for MG adsorption on BaTiO₃

The magnitudes of ΔH° and ΔS° can be derived from the slope and intercept of the (ln Kc versus 1/T) plot (Van’t Hoff plot) exhibited in Fig. 10.

Thermodynamic variables are listed in Table (4). The magnitudes of ∆G° remained negative across the temperature range investigated, indicating that the process of adsorption was spontaneous and thermodynamically favorable. The magnitude of ∆H° was positive, indicating that the adsorption process is endothermic in nature. The positive magnitude of ∆S° was a result of the greater randomness at the BaTiO₃/MG solution interface because of BaTiO₃ affinity towards MG dye. The chemisorption process of adsorption is again verified by the positive magnitude of ΔH°, which is higher than 40 kJ/mol.

**Table 4:** Variables of thermodynamic of MG adsorption by BaTiO₃ at different temperatures

T (K)	∆G° (kJ/mol)	∆H° (kJ/mol)	∆S° (kJ/mol K)
298	-27.867	67.113	0.316
303	-28.964		
308	-30.024		
313	-31.715		
318	-33.685		
323	-35.909		

#### Desorption and reusability

**Fig. 11:**
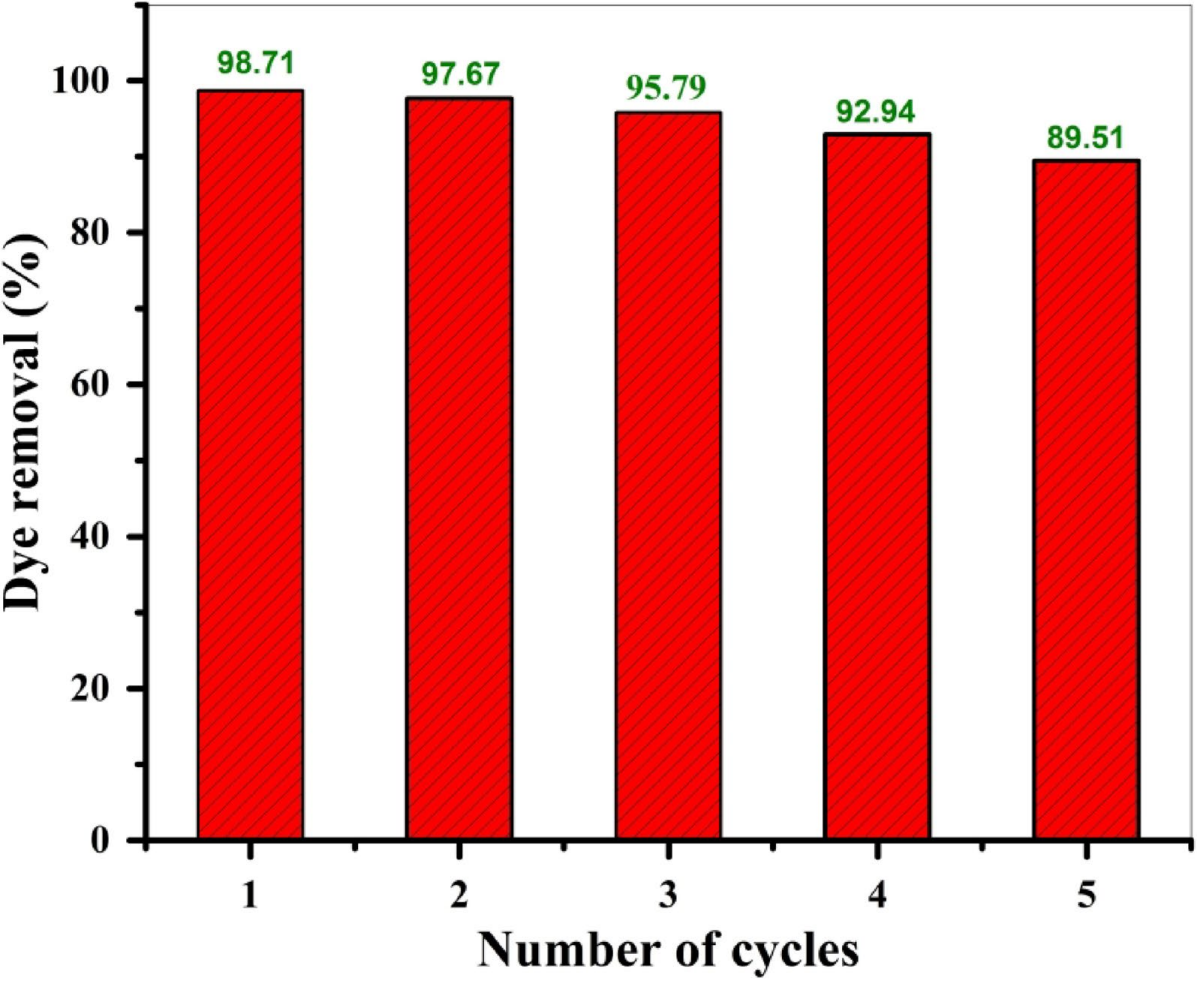
Percentage removal of MG dye using the recycled adsorbent compared with a fresh one: (Conditions: initial dye concentration = 500 mg/L, adsorbent dose = 10 mg, volume = 10 mL, temperature = 25 °C, equilibrium time = 330 min, pH = 5)

The reusability of BaTiO₃ for the adsorption of MG was investigated because it is crucial for the adsorbent’s economic and practical application. Five successive adsorption-desorption cycles were carried out under the optimized conditions (C_o_ = 500 mg/L, adsorbent dosage = 10 mg, volume = 10 mL, equilibrium time = 330 min., pH = 5) at room temperature (T = 25 °C), and the MG dye molecules were desorbed from the BaTiO₃ adsorbent using absolute ethanol, then rinsed with deionized water and dried for 1.0 h. at 70 °C before being used for another adsorption cycle. As exhibited in Fig. 11, the MG removal percentage slowly reduced from 98.71% to 89.51% after the 5th cycle. Therefore, MG removal using BaTiO₃ remained significant even after the fifth cycle.

#### Comparison with other adsorbents

Various adsorbents for MG dye and their adsorption capacities are displayed in Table 5. It is obvious from the table that BaTiO₃ has a superior maximum adsorption capacity (495.15 mg/g) compared to others; this is due to its mesoporous structure and surface chemistry (electrostatic attraction and chemisorption mechanism). So BaTiO₃ is an efficient adsorbent to remove MG dye from aqueous media.

**Table 5:** Comparing adsorption capacity of BaTiO₃ with other adsorbents

Adsorbent	q_m_ (mg/g)	References
BaTiO₃ powder	495.15	This work
Activated sintering process red mud	336.4	[41]
Teak leaf litter powder	333.33	[42]
Mn-doped CuO-NPs‐AC	320.51	[43]
ZnO	310.5	[44]
TiO₂/γCD NPs	244.0	[45]
CONP	238.1	[46]
Commercial powder activated carbon	222.22	[47]
Dead leaves of plane tree	97.09	[35]
SnO₂	216.9	[44]
Au-NPs-AC	164.57	[48]
NiO	158	[49]
SnO₂ NPs-AC	142.87	[50]
Chitosan beads	93.5	[51]
Walnut shell (WS)	90.8	[52]
Activated carbon-CoFe₂O₄ composites	89.3	[53]
NONP	87.72	[54]
Bivalve shell-Zea mays L husk leaf	81.5	[55]
Graphite oxide/polyurethane (GO/PU)	68.82	[56]
Rattan sawdust	62.71	[57]
Degreased coffee bean	55.3	[58]
Pineapple leaf powder	54.64	[59]
SrTiO₃ nanoparticles	35.9	[60]

#### Real samples

**Fig. 12:**
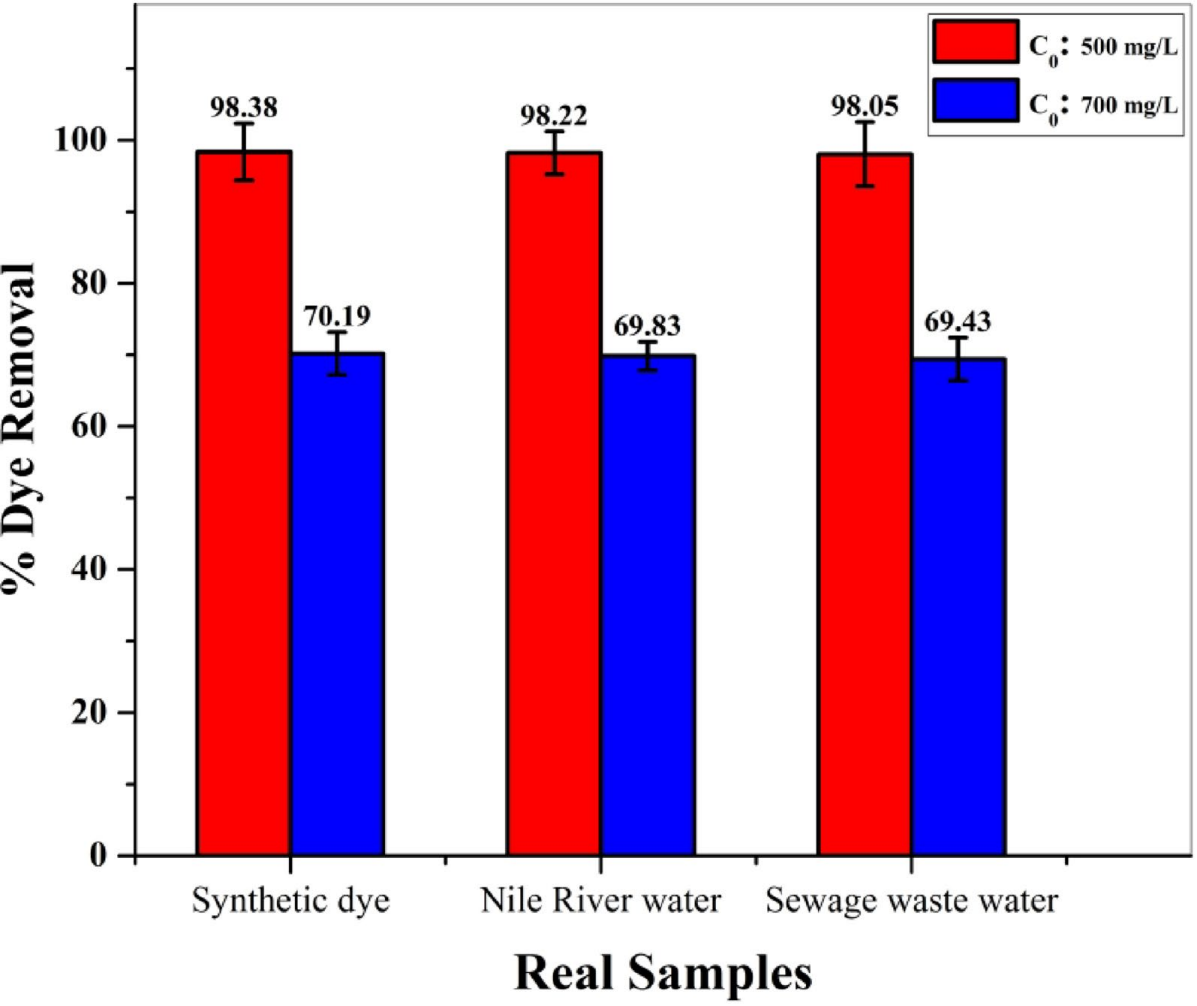
the percentage of simultaneous removal for MG dye in Real samples: (*Conditions*: adsorbent dose = 10 mg, volume = 10 mL, temperature = 25 °C, equilibrium time = 330 min, pH = 5)

To investigate the method’s efficiency and usability for real samples, it was utilized to remove and measure MG in different water samples, for example, Nile River water, sewage wastewater, and synthetic dye effluent. The samples were spiked with MG at the initial concentration of dye (500 and 700 mg/L), and the process was proceeded under the optimal conditions (adsorbent dosage: 10 mg, volume: 10 mL, pH: 5, contact time: 330 min.) at room temperature (298 K). The removal efficiencies at 500 and 700 mg/L of MG dye were quite similar, with values between 98.38 and 98.05% and 70.19–69.43%, respectively, revealing negligible changes with different water samples (Fig. 12). This means that BaTiO₃ is a promising adsorbent in the applications field.

**Table 6:** Characteristic of environmental water samples analysis

Nile river water		Sewage wastewater		Synthetic dye effluent	
Items	mg/L	Items	mg/L	Items	mg/L
TDS	315	TDS	1005	NaCl	15
Turbidity (NTU)	15.24	TSS	19	Na_2_SO_4_	15
BOD	10	BOD_5_	52	CH_3_COONa	15
COD	6	COD	66	Na_2_CO_3_	15
DO	6.27	DO	6.4	Na_2_HPO_4_	15
NO_2_	0.532	NO_2_	25	MgSO_4_	15
NO_3_	3.156	NO_3_	0.3	CaCl_2_	15
PO_4_	0.960	PO_4_	3.2	KCl	15
NH_3_-N	0.031	NH_3_-N	3	-	-
Oil & grease	NIL	Oil & grease	5.8	-	-

#### Proposed adsorption mechanism

Numerous mechanisms involving adsorbent-adsorbate interactions are studied in the MG dye adsorption process on the BaTiO₃ surface. Adsorbent-adsorbate binding is dependent on various variables, including surface characteristics, pH of solutions, functional groups, and dye molecular structure. The proposed interaction mechanism between MG and BaTiO₃ may be a combination of electrostatic forces and weak interactions (Van der Waals force). The pH_ZPC_ value for BaTiO₃ is 2.3. Above pH 2.3, the BaTiO₃ surface was negatively charged due to the existence of oxygen atoms. The cationic MG dye was attracted electrostatically by the negative charge on the BaTiO₃ surface. Considering that the kinetic results were best suited to the second-order model and for isotherms were Langmuir model, it could be indicated that the mechanism of MG adsorption is chemisorption as a monolayer on BaTiO₃ surface. FTIR analysis yields more evidence. After MG adsorption, additional peaks established at 1316 cm⁻¹ and 1604 cm⁻¹, representing C-N stretching and aromatic C = C stretching vibrations, respectively. Furthermore, some BaTiO₃ peaks shifted slightly (e.g., 439 → 441 cm⁻¹, 1082 → 1074 cm⁻¹, 1448 → 1447 cm⁻¹, and 1772 → 1770 cm⁻¹) and exhibited changes in intensity. These alterations in the spectrum confirm the interaction between the functional groups of MG and the surface of BaTiO₃, indicating chemisorption in the adsorption process.

## Conclusion

In this study, BaTiO₃ powder was easily synthesized, characterized, and employed as an adsorbent to remove MG dye from aqueous solutions. The characterizations (XRD, FTIR, SEM, TEM, EDX, and BET test) were executed to estimate the chemical composition, crystallinity, morphology, and porosity of BaTiO₃ adsorbent. The various parameters affecting the adsorption efficiency, such as pH, initial dye concentration, adsorbent amount, contact time, temperature, and ionic strength, on the removal of MG were optimized as follows: pH (5–10), initial concentration of dye (500 mg/L), adsorbent dosage (10 mg), and contact time (330 min). The adsorption capacity of BaTiO₃ rises as temperature rises and reduces as ionic strength rises. The maximum adsorption capacity for BaTiO₃ can attain 495.15 mg/g. The adsorption process obeyed a pseudo-second-order kinetic model and Langmuir isotherm. Thermodynamic results suggest that the adsorption is endothermic in nature and spontaneous. The MG adsorption on BaTiO₃ powder is a chemisorption process evident by FTIR analysis and kinetic and thermodynamic studies. BaTiO₃ is stable and can be utilized again for up to five cycles with a slight decrease in adsorption efficiency. The study indicated the usefulness of BaTiO₃ to remove MG dye from contaminated wastewater. This work introduces an economical, environmentally friendly, scalable, and highly efficient adsorbent. Future work should focus on modifying the BaTiO₃ surface and evaluating adsorption performance under composite of BaTiO₃ with metal oxide.

## Data Availability

The datasets used or analyzed during the current study are available from the corresponding author upon reasonable request.
